# Learning-Based Autonomous UAV System for Electrical and Mechanical (E&M) Device Inspection

**DOI:** 10.3390/s21041385

**Published:** 2021-02-16

**Authors:** Yurong Feng, Kwaiwa Tse, Shengyang Chen, Chih-Yung Wen, Boyang Li

**Affiliations:** 1 Department of Mechanical Engineering, The Hong Kong Polytechnic University, Kowloon 999077, Hong Kong; yurong.feng@connect.polyu.hk (Y.F.); sky.tse@polyu.edu.hk (K.T.); shengyang.chen@connect.polyu.hk (S.C.); chihyung.wen@polyu.edu.hk (C.-Y.W.); 2 Interdisciplinary Division of Aeronautical and Aviation Engineering, The Hong Kong Polytechnic University, Kowloon 999077, Hong Kong

**Keywords:** UAV, autonomous inspection, object detection, deep learning

## Abstract

The inspection of electrical and mechanical (E&M) devices using unmanned aerial vehicles (UAVs) has become an increasingly popular choice in the last decade due to their flexibility and mobility. UAVs have the potential to reduce human involvement in visual inspection tasks, which could increase efficiency and reduce risks. This paper presents a UAV system for autonomously performing E&M device inspection. The proposed system relies on learning-based detection for perception, multi-sensor fusion for localization, and path planning for fully autonomous inspection. The perception method utilizes semantic and spatial information generated by a 2-D object detector. The information is then fused with depth measurements for object state estimation. No prior knowledge about the location and category of the target device is needed. The system design is validated by flight experiments using a quadrotor platform. The result shows that the proposed UAV system enables the inspection mission autonomously and ensures a stable and collision-free flight.

## 1. Introduction

Electrical and mechanical (E&M) devices are crucial to the efficient operation of the public transportation system. They need to be maintained periodically to ensure safety and serviceability. Traditionally, E&M devices are inspected by certified operators, which can be costly, labor-intensive, and hazardous in locations that lack accessibility. With the rapid advancement of computer vision technologies, unmanned aerial vehicles (UAVs), especially quadrotors, have been applied to many aspects recently, such as aerial photography [1], device inspection [2,3,4], search and rescue [5], and so on. Benefitting from their mobility, flexibility, and multi-functionality, UAVs have shown significant potential as a cost-efficient solution for routine visual inspection.

A variety of methods for UAV inspection have been proposed in recent years. Maethe et al. [6] proposed a vision-based method to inspect railway infrastructure. Several traditional computer vision techniques were used to detect objects, and an Extended Kalman Filter (EKF) method was applied to estimate the position of objects. However, this approach suffered from inaccurate distance measurement, which may not be robust enough for real-world applications. Using a hexacopter equipped with a manipulator, Steich et al. [7] demonstrated the inspection of tree cavities semi-autonomously. They used a contour detection algorithm to detect tree cavities and the Kalman Filter to generate continuous position estimation. However, the small-scale features in images could potentially result in detection failure of tree cavities. Nikolic et al. [8] presented a UAV system for the inspection and 3-D reconstruction of an industrial boiler. Their quadrotor was equipped with an active illumination module to detect structural damages of the boiler walls at short range. Meanwhile, a 3-D structure scan of the boiler was recorded using a stereo camera. The aforementioned systems or methods are composed of multiple functionalities together to support different inspection missions. Most of them rely on traditional computer vision techniques in image post-processing for object recognition, which are generally not robust enough for various geometric or photometric changes. In this paper, we opt to apply the learning-based approach—the deep neural network (DNN) method—to perceive various target objects in an unknown environment. The “learning-based” refers to the object detection method embedded in the perception module, which is a supervised learning algorithm.

Despite these works, some challenges still prohibit the broad application of device inspection by UAVs. First, some E&M devices are deployed in potentially confined areas such as in tunnels or beneath bridges. It is challenging for a quadrotor to gain reliable state estimation in the absence of GPS signals. Second, the inspection task carried out manually is usually tedious and time-consuming. With the support of UAVs, although aerial photography can be performed by the manual flight and viewing images of the target devices from the camera, the whole pipeline of maneuvering the UAV is still complicated and energy-consuming for the pilots.

To address all these issues, in this work, we focus on the autonomous inspection quadrotor system without requiring prior knowledge of the target device and environment. To ensure the quadrotor system is fully autonomous, we require all computation to occur onboard in real-time without external communication or human interference. Therefore, we designed the UAV system for tasks including perception, localization, and path planning using only onboard sensors. The contributions of this work are summarized as follows:

1. A learning-based 3-D object state estimation method is proposed. We exploit a learning-based object detector to recognize the target devices. A filter-based object state estimation method is proposed to estimate the target device state (3-D position and collision). No prior knowledge of the category of the devices is needed.
2. A geometric constrained path planning method is designed to navigate the quadrotor system autonomously. Our path planning module shows promising performance to eliminate human involvement in inspection missions.
3. Visual-inertial odometry (VIO) is integrated into the system to provide accurate pose estimation in GPS-denied environments. All components in the system are released as open-source packages.
4. We present a set of experiment results in different scenarios to validate the performance of our system.

The rest of this paper is structured as follows. We discuss relevant literature in Section 2 and introduce the overall system architecture in Section 3. The implementation details of the whole system are provided in Section 4. Section 5 describes the experiment and results. Finally, the paper is concluded with a discussion and possible future research directions in Section 6. We released our implementation codes of deep learning models, dataset, and path planning algorithm as the Supplementary Materials.

## 2. Related Work

For real-time autonomous inspection in GPS-denied environments, the quadrotor system must process perception, localization, and path planning problems on computational-resource-limited (speed and memory) onboard computers. Each of these topics comprises a range of literature. Here, we focus on research that directly impacts our system design and methodology.

### 2.1. Object Detection

Object detection has been well studied by many researchers over the last few years and has advanced considerably. Some detectors are optimized for GPU platforms, such as VGG [9], ResNet [10], or DenseNet [11], whereas some detectors are running on CPU platforms with a backbone, such as MobileNet [12,13]. A contemporary object detector consists of two parts, a pre-trained *backbone* to extract visual features and a *head* to predict classes and bounding boxes. The *head* can be categorized into the one-stage and two-stage object detectors. R-CNN series [14] are the most common two-stage detectors, including fast R-CNN [15], faster R-CNN [16], and R-FCN [17]. Two-stage detectors are more accurate than one-stage methods, but most cannot achieve real-time performance. For one-stage object detectors, You Only Look Once (YOLO) series [18,19,20], Single Shot MultiBox Detector (SSD), and RetinaNet [21] are the popular networks based on the anchor mechanism. Some researchers inserted layers between the backbone and the head to collect feature maps from different scales, which is called the *neck*. Object detectors adopting *neck* mechanisms, such as feature pyramid network (FPN) [22] and path aggregation network (PAN) [23], have semantically strong capabilities to improve detection accuracy on small-sized objects. For aerial inspection missions, the state-of-the-art one-stage object detector YOLOv4 has been chosen as our perception solution since it provides a good balance between accuracy and speed on the resource-limited onboard computer.

### 2.2. Localization

A range of methods has been pursued to solve the pose estimation problem in GPS-denied environments. Most previous approaches use LiDAR- [24] or camera-based methods [25,26,27] to perceive the environment. For aerial inspection scenarios, a low-cost, small-size, camera-based solution is preferable. In recent years, the stereo-visual-inertial solution has been gaining popularity. Compared with the monocular solution, the inertial measurement unit (IMU) can estimate the pose between consecutive visual frames to increase robustness. When visual tracking is lost, IMU can maintain the pose output during a short period. The stereo-visual-inertial solution fuses more measurement in the pose estimation process, which leads to better accuracy. Another feature of the stereo visual-inertial solution is that the depth information can be extracted directly from stereo images without any motion. Thus, the system scale is consistent, and the initialization can be achieved in one shot. These works can be mainly categorized into filter-based methods and optimization-based methods. Most filter-based methods leverage the Extended Kalman Filter (EKF), where camera and IMU measurements are treated as independent modules. MSCKF is a popular EKF based method [28,29]. The authors maintained multiple previous camera poses and exploited visual measurements from the same feature in different camera views. Filter-based methods are dependent on timely coming measurements in the filter procedure. Therefore, a particular sequence sorting mechanism should be developed to place measurements from multiple sensors in order. In EKF-based approaches, using IMU for state propagation is the most straightforward method.

Optimization-based methods, also known as bundle adjustment (BA), use the graph data structure to store and optimize the camera pose and measurements. In the structure of the pose graph, each vertex represents camera pose and landmarks. Edge is defined by adjacent vertex in terms of visual measurement and propagated IMU measurement. For a time-synchronization problem, optimization-based methods have many advantages when compared to filter-based methods. First, measurements from different sensors can be easily handled in the graph data structure. Second, optimization-based methods also outperform filter-based methods in terms of accuracy performance but at the cost of computation overhead. In the current quadrotor system, our previous work feedback loop based visual-inertial SLAM (FLVIS) [30] is adopted for pose estimation. FLVIS is a state-of-the-art pose estimation solution and has shown great competence compared with other benchmarks in GNSS-denied environments.

### 2.3. Quadrotor Path Planning

Path planning is essential to generate a safe and physically feasible trajectory for the quadrotor system. The problem of path planning is mainly addressed by a front-end and a back-end independently. The front-end planning searches for an available path from the start point to the target point within a given map. The general method of path finding is based on graph search algorithms. The representative two methods of the search-based methods are the A* [31] and jump point search (JPS) algorithms [32]. The A* algorithm serves as a globally optimal solution, and search efficiency can be reduced by introducing a heuristic function. However, due to path symmetries, A* algorithm wastes time on expanding unnecessary nodes. The JPS algorithm is an effective technique for eliminating path symmetries, which can be described as a combination of the original A* with neighbor-pruning rules. In addition, Arzamendia et al. [33] modeled the path planning problem into a constrained Travelling Salesman Problem (TSP). A genetic algorithm was adopted to search for the maximum coverage area of the inspected area. Peralta et al. [34] presented a local path planning method called Updating the Fast Marching Square method (uFMS) to adapt to various dynamic environments. The experiment results showed that their proposed method could generate a smoother curve and achieved a higher security level. For back-end planning, the purpose is to generate and optimize a time-based trajectory for vehicles to execute. Mellinger et al. [35] proposed a minimum snap trajectory generation algorithm. The trajectory is formulated into piecewise polynomials and optimized using a quadratic program (QP) solver. Charles et al. [36] proposed a closed-form minimum snap trajectory generation method using an unconstrained quadratic program. Their contribution shows that the solution of polynomial trajectory is numerically stable and efficient. As our work focused on inspecting E&M devices, we did not consider aggressive flight. Our method generates a piecewise trajectory based on the estimated position of the detected target. And a high-level decision making is used to avoid collision during flight.

### 2.4. Vision-Based UAV Inspection System

In recent years, some state-of-the-art UAV systems have been published and demonstrated the vision-based inspection pipeline. Steich et al. [7] successfully used a hexacopter to inspect tree cavities. They used a clustering algorithm “k-means” to segment the boundary of tree cavities from the background by depth. Then, a contour detection algorithm combined with ellipse assumption was applied to detect tree cavities. However, their system relied on feature descriptor to detect salient repeatable features, which is susceptible to small scale objects and environment changes. Thus, the UAV system needs to be operated by a human pilot to approach the tree cavity. Besides, the ellipse assumption provided prior knowledge to the detection module and constrained its application to single target detection with a simple geometric shape. In this work, the proposed system overcomes detection failures incurred by the small metric scale, and multiple target inspection missions could be conducted with much ease. Fehr et al. [37] proposed a Teach-and-Repeat (T&R) scheme that allows a UAV to follow a previously traversed route for inspecting industrial facilities periodically. Their method relied heavily on robust visual-inertial-odometry (VIO) and a mapping framework with expensive computational overhead costs. Gao et al. [38] proposed a similar system focusing on generating a smooth trajectory, which proved to be an energy-saving solution. However, the global map for repetitive inspection route is vulnerable to environmental change. In other words, even if the environment changes by a small margin, the map must be rebuilt. Compared with these works, our method uses a versatile and flexible framework regardless of global mapping computation. Using robust learning-based detection, our system can percept multiple objects from a medium distance without human involvement.

## 3. System Overview

**Figure 1:**
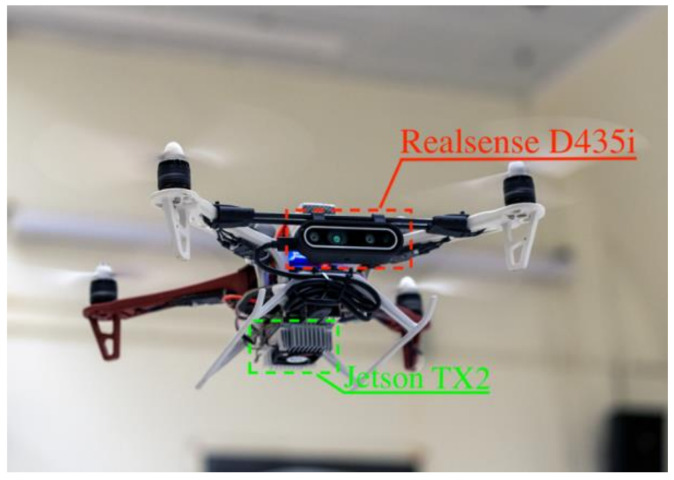
The unmanned aerial vehicle (UAV) inspection system during flight.

For visual inspection purposes, the UAV requires sensors to stabilize and navigate the vehicle itself and detect target devices. We assembled a UAV system equipped with an Intel RealSense D435i stereo camera and a Jetson TX2 onboard computer. The hardware platform of our quadrotor system is shown in Figure 1.

**Figure 2:**
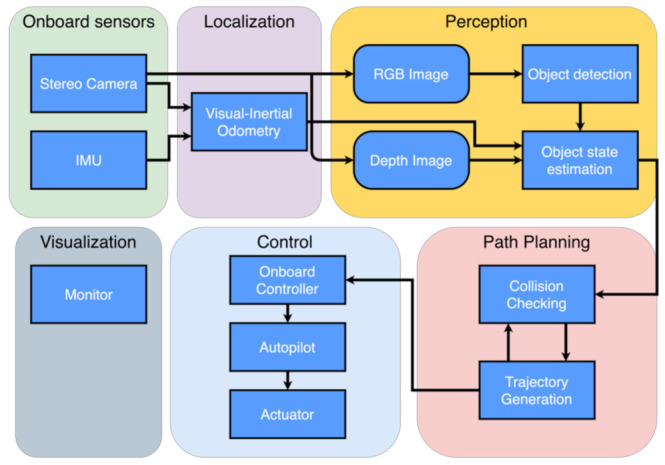
The software architecture of our UAV inspection system.

The software architecture of our inspection UAV system includes four parts. The primary functionality modules of localization, perception, and path planning are running on the onboard computer while visualization is running on an offboard laptop. An illustration of the software architecture is shown in Figure 2. The localization modules build the foundation of our quadrotor inspection system. In this study, we use the visual-inertial odometry (VIO) framework, FLVIS, developed by our group to obtain accurate pose estimation without relying on the GPS signal.

The perception module detects target devices for inspection and feeds the states of objects into the path planning module. The essential step in sensing the target device is to identify the device and estimate its position in an unknown environment. Therefore, the problem can be divided into sub-problems of 2-D object detection and 3-D object state estimation. The captured RGB images are processed by the object detector YOLO, resulting in object regions represented by 2-D bounding boxes. With consecutive regions of interest (ROI), we utilize a filter-based object state estimation method to obtain the 3-D position of the detected object. The 2-D bounding boxes regressed by object detector and depth measured from the stereo camera are tightly coupled to solve the 3-D position of objects in the world frame. Meanwhile, the background pixels are used to define the collision state of the object.

Based on the state of objects, a flight path is generated by the path planning module. To carry out inspection missions, the path planning module builds a finite state machine model for our quadrotor system. The model defines the whole pipeline of an inspection mission, and the state of the vehicle can be switched sequentially. In the search stage, the path planning module commands yaw motion to search objects in space. Then, in the check stage, the path planning module generates a real-time spatial trajectory to inspect each object in order. Finally, trajectory tracking is handled by the autopilot.

Though the inspection work can be conducted autonomously by the quadrotor system, it is still straightforward and convenient for researchers to test the quadrotor system and monitor the whole process of an inspection mission. The visualization module is developed in ROS (robot operating system) environment. The primary function of the visualization model is two-fold: detected object visualization in 3-D space and a 30 Hz real-time video streaming of the front-facing camera.

## 4. Object Detection and State Estimation

### 4.1. Object Detection

The perception of the target devices starts with the 2-D object detection, which will generate a 2-D bounding box for every E&M device in aerial images. The 2-D bounding box of the object is used to represent the object regions, and consequently, the regions of interest in depth images are used for object state estimation. For each object region, the inner ROI will be used for position estimation. In contrast, the outer ROI will be used for collision estimation. If the multiple-object detection is needed, several independent 2-D bounding boxes will be generated simultaneously. The corresponding object regions will be processed by independent ROI generation threads, where each object’s state will be estimated simultaneously. To achieve high accuracy and real-time performance in our UAV system, we adopt YOLO-v4 as the 2-D object detector.

#### 4.1.1. 2-D Bounding Box Prediction

**Figure 3:**
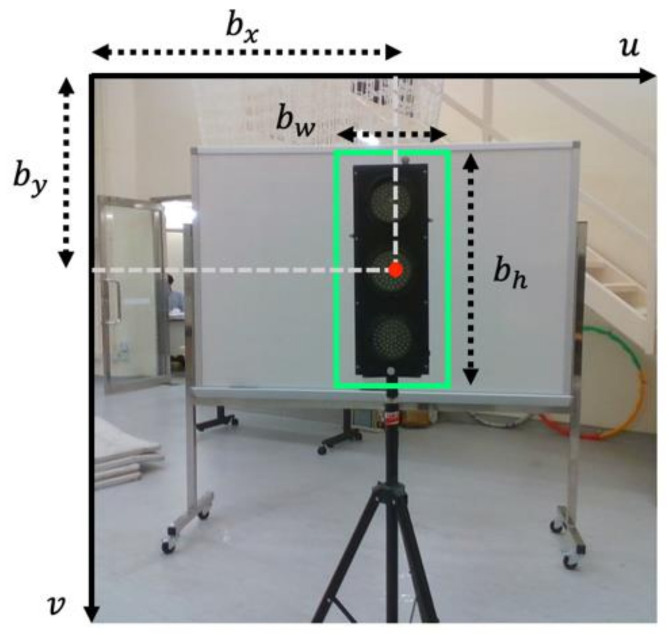
2-D bounding box prediction in image plane (green rectangle represents the 2-D bounding box).

YOLO is a supervised-learning algorithm to predict the 2-D location of objects in an image. It splits the input image into grid cells and predicts multiple bounding boxes for each grid cell. For each cell, an anchor-based method is used to maximize the intersection over union (IOU) between the ground truth box and the bounding box [19]. Instead of using anchors designated with the fixed scale and aspect ratio in Faster R-CNN [16], YOLO uses the k-means clustering to generate corresponding aspect ratios of anchor boxes in different feature maps. For each bounding box prediction in the image plane, YOLO predicts four parameters: $t_{x}$, $t_{y}$, $t_{w}$, $t_{h}$. The prediction principle is that if the cell is offset from the top left corner from the image by $\left(c_{x},c_{y}\right)$ and the anchor box has width $p_{w}$ and height $p_{h}$, then the bounding box prediction is computed by [20]:

(1) $$b_{x}=\sigma \left(t_{x}\right)+c_{x}\;b_{y}=\sigma \left(t_{y}\right)+c_{y}\;b_{w}=p_{w}e^{t_{w}}\;b_{h}=p_{h}e^{t_{h}},$$

where $b_{x}$ and $\;b_{y\;}$ are the center coordinates, $b_{w}$ and $b_{h}$ the width and height of the predicted bounding box, respectively, and σ is the sigmoid function applied to constrain the offset range between 0 and 1. Figure 3 shows an illustration of the bounding box predicted by YOLO.

#### 4.1.2. Training Dataset Preparation

**Figure 4:**
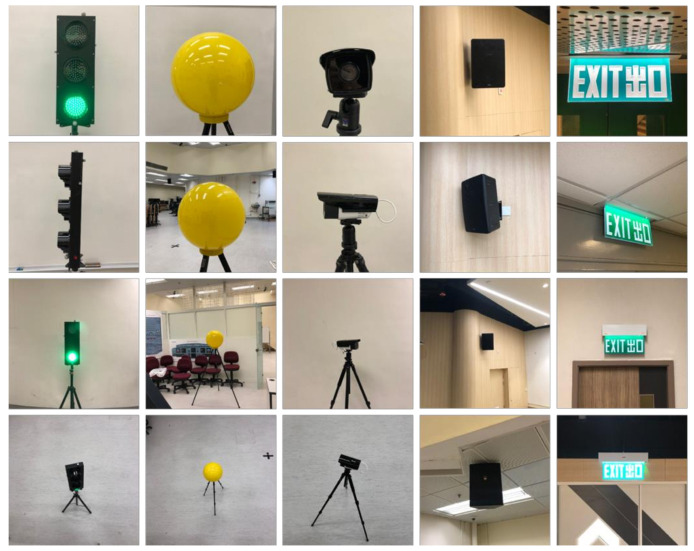
Composite image samples of the training dataset.

A series of sample devices, including traffic light, bulb, exit sign, amplifier, and closed-circuit television (CCTV), were adopted as target devices in the experiments to simulate the inspection targets. We prepared a customized medium-scale dataset, including around 20,000 images in total, which was composed of 2000 images per class as well as 10,000 empty background images. The dataset contains images with different illumination conditions, view angles, distances from objects, scales, and resolutions, as shown in Figure 4. All images were captured on the campus of the Hong Kong Polytechnic University. The training result will be further detailed in Section 6.1.

### 4.2. Region of Interest Generation

Followed by object detection, the region of interest (ROI) generation involves segmenting the proper region for 3-D object position estimation and collision estimation. The input of ROI generation is the aligned depth image and RGB image with the same resolution of 640 × 480. Then, RGB images are used for object detection to predict 2-D bounding boxes in each frame. Considering that the 2-D bounding box may include background pixels, we did not directly utilize object regions in depth images; we used two scaled regions in the depth image to represent the spatial information of object and background separately.

Given the 2-D bounding box predicted by the object detector, the object region can be represented by the center coordinate, width, and height of the bounding box from Equation (2):

(2) $$S_{o}=\left[\begin{array}{cccc} b_{x} & b_{y} & b_{w} & b_{h} \end{array}\right].$$

**Figure 5:**
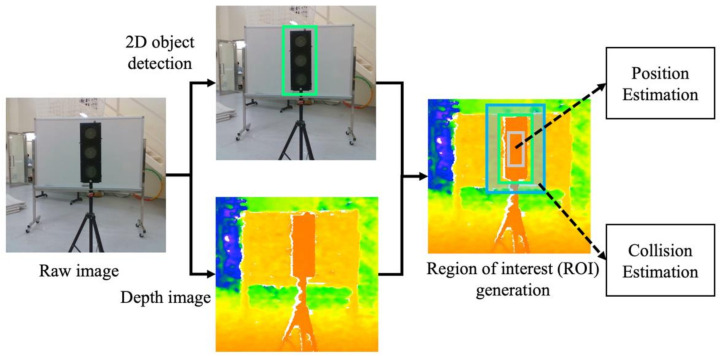
Region of interest generation. Green rectangle: 2-D bounding box $S_{o}$; Grey rectangle: inner region $S_{in}$; Blue rectangle: outer region $S_{out}$.

We assume the inner region and outer region have the same aspect ratio as the object region. Thus, the inner region $S_{in}$ and outer region $S_{out}$ can be defined by two different scale factors α and β as follows:

(3) $$S_{in}=\left[\begin{array}{cccc} b_{x} & b_{y} & \alpha b_{w} & \alpha b_{h} \end{array}\right],\;S_{out}=\left[\begin{array}{cccc} b_{x} & b_{y} & \beta b_{w} & \beta b_{h} \end{array}\right],$$

where  α and β determine the number of pixels in the ROI to be estimated. We set α=0.3 and β=1.2 as empirical values in our experiments. The ROI used for object position estimation and collision estimation can be formulated as:

(4) $$S_{obj}=S_{in},\;S_{background}=S_{out}⊖S_{o},\;$$

where $S_{obj}$ and $S_{background}$ denote the ROI for object position estimation and collision estimation, respectively; ⊖ refers to the difference operation of the two areas. The workflow of ROI generation is shown in Figure 5.

### 4.3. Object State Estimation

The objective of this module is to recover the 3-D object position and check the collision-free state from the ROI of sequential depth images. Before doing object state estimation, we already have the ROI in depth images. In order to extend 2-D object detection to 3-D object state estimation, we use 3-D stereo reconstruction to recover the position of detected objects.

#### 4.3.1. Object Position Estimation

**Figure 6:**
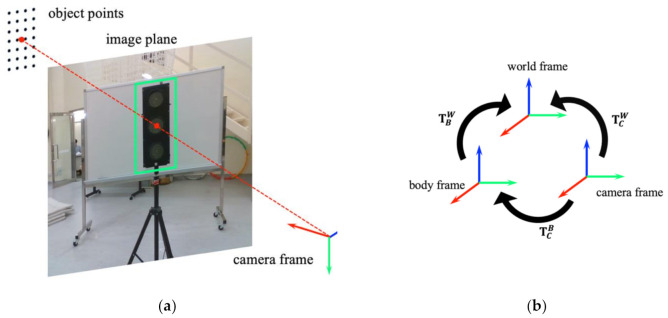
An illustration of 3-D object position estimation: (**a**) 3-D reconstruction of point in camera frame; (**b**) Coordinate frames relationship in the system.

Since the stereo camera only measures the distance between the surface of the 3-D object and the camera, we assume the object dimension is known as the sphere’s radius or the rectangular cuboid’s length. Here the depth of a point in a depth image is increased by the object dimension s as:

(5) $${\tilde{\lambda }}_{i}=\lambda_{i}+s,$$

where $\lambda_{i}$ is the depth value of each pixel in the corresponding ROI $S_{obj}$ provided by the depth image. With the obtained depth of every point, we now project the 2-D points into 3-D space using the perspective projection model in homogeneous coordinates:

(6) $${\tilde{\lambda }}_{i}\left[\begin{array}{c} u_{i}\\ v_{i}\\ 1 \end{array}\right]=K\cdot \left[\begin{array}{c} X_{i}^{C}\\ 1 \end{array}\right],$$

where K is the intrinsic camera matrix, $X_{i}^{C}$ is the Cartesian coordinates of each point in the camera frame, $u_{i}$ and $v_{i}$ are the corresponding pixel coordinates of the projection in the depth image. The point $X_{i}^{C}$ is then transformed into the world frame by applying:

(7) $$\left[\begin{array}{c} X_{i}^{W}\\ 1 \end{array}\right]=T_{B}^{W}T_{C}^{B}\left[\begin{array}{c} X_{i}^{C}\\ 1 \end{array}\right],\;T_{B}^{W},T_{C}^{B}\in SE\left(3\right),$$

where $T_{B}^{W}$ is the transformation representing the pose of the body frame with respect to the world frame provided by the localization module, and $T_{C}^{B}$ is the transformation from the camera frame to the body frame. The coordinate frame relationship is illustrated in Figure 6b. Then, the 3-D object position $X^{W}$ can be estimated by the mean value of all object points in the same ROI (Figure 6a).

#### 4.3.2. Infinite Impulse Response Filter

Once the 3-D position of an object has been estimated, further processing is required to provide valuable information to the path planning module because: (1) the raw depth measurement of the stereo camera has large amounts of high-frequency noise, and (2) the depth measurement by the stereo camera has a large error, especially at large distances. We applied an infinite impulse filter (IIR) to the object’s position estimate as the input for the measurement update. Since each frame is independently observed by the camera, the depth value obtained can be assumed to be a random variable following Gaussian distribution. So, the current estimated position is formulated as:

(8) $${\bar{X}}_{k}^{W}=\theta \cdot X_{k}^{W}+\left(1-\theta \right)\cdot {\bar{X}}_{k-1}^{W},$$

where ${\bar{X}}_{k}^{W}$ denotes the current estimated position, $X_{k}^{W}$ is the current measured position, ${\bar{X}}_{k-1}^{W}$ is the previously estimated position, and θ is the IIR filter parameter. In all our experiments, we set θ=0.9, which has been selected empirically and seems satisfactory. Through the IIR filter, the measurement error will converge to a neglectable small value, the results of which are detailed in Section 6.2.

#### 4.3.3. Collision Estimation

An essential operation in UAV inspection is collecting visual data of targets through aerial vehicles. Considering that the E&M devices are installed with different configurations, collisions may occur if the target has close obstacles behind it. First, we estimate the average distance between obstacle and camera as $\bar{d}$ and the average distance between the object and camera as $\bar{\lambda }$. Then, we use the logic elaborated in Algorithm 1 to estimate the object’s collision state.
**Algorithm 1:** Collision state estimation. **Notation**: Circle radius R, Object $o_{i}$, Object set O, Object dimension $s_{o}$, UAV dimension $s_{u}$, Object depth $\bar{\lambda }$, Environment depth $\bar{d},$ Boolean object collision state collsion_state.**Input:**O**for**$o_{i}$ in O**if**$R<\bar{\lambda }$**then****if**$R>s_{o}+s_{u}\;$&& $R<\bar{d}-s_{o}\;$**then**collsion_state⟵safe   **else**collsion_state⟵collision**end if****else**collsion_state⟵collision**end if**$o_{i}⟵O$.**next**()**end for****return**O

The algorithm is initialized by checking the first detected object. Then, all the detected objects in the single frame are checked sequentially until the last one. For a detected object, if the distance from its surface to the camera ($\bar{\lambda }$) is smaller than the predefined circle radius (R), it means that the UAV is flying too close to the object. In this case, the object collision state (collsion_state) will be updated as collided. If the circle radius (R) exceeds the total dimension of the object and UAV ($s_{o}+s_{u}$) and does not exceed the difference between the distance of background and object ($\bar{d}-s_{o}$), it means that the UAV can track a circle around the target object without collision. As such, the object collision state is updated as safe. In engineering implementation, the detected objects are stored in a sequential queue data structure and checked by using the **next**() function iteratively. The object collision state is implemented by a Boolean data type, denoting a binary state safe or collision.

## 5. Inspection Path Planning

The path planning module of the whole system is leveraged to navigate the quadrotor through the whole inspection mission. Considering multiple target devices to be inspected, the path planning module consists of two parts: a finite state machine (FSM) model for governing the behavior of the UAV and a geometrical method to find waypoints for a circle trajectory, which is waypoints generation.

### 5.1. Finite State Machine

**Figure 7:**
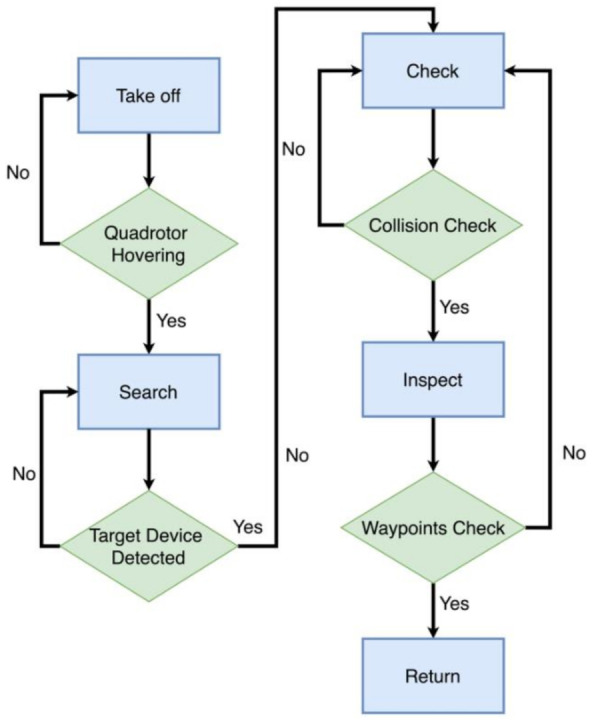
Flow chart of our finite state machine (the state of the vehicle is marked with blue boxes).

The state machine module manages the behavior of the quadrotor during the entire inspection mission. It has four states, namely: takeoff, search, inspect, and return. Figure 7 depicts the state machine with its states and the respective transitions triggered by events. In the following paragraphs, we describe each of the states in more detail.

1. *Takeoff:* We initialize our visual inertial-odometry framework FLVIS to maintain full state estimation. At this point, our quadrotor launches from the ground and is commanded to reach a hover state within a given period of time. Once the vehicle is hovering, we switch the state machine to the *Search* mode.
2. *Search:* The quadrotor explores an area by yawing from –90° to +90° at a given height. When the front-facing stereo camera detects the target devices, the vehicle autonomously computes the states of objects (Section 4.3) and generates waypoints. The waypoints generation method will be introduced in Section 5.2. This mode ends when the UAV yaws back to the original steering angle again and enters the *Check* mode.
3. *Check:* In this mode, the quadrotor heads to the target and approaches the target device by flying to the waypoint. After the quadrotor reaches the waypoint, it is commanded to reach a hover state and check the collision status of the target described in Section 4.3.3. If the UAV status is safe, the state machine is switched to *Inspect* mode.
4. *Inspect:* In this mode, the quadrotor tracks a circular trajectory around the object at a given angular velocity. After the circle trajectory is finished, the state machine is switched back to *check* mode. If all the detected objects have been inspected, the state machine automatically transitions to the *Return* mode.
5. *Return:* The quadrotor is commanded to return to the home position and lands at a constant vertical velocity.

### 5.2. Generation of Waypoints

**Figure 8:**
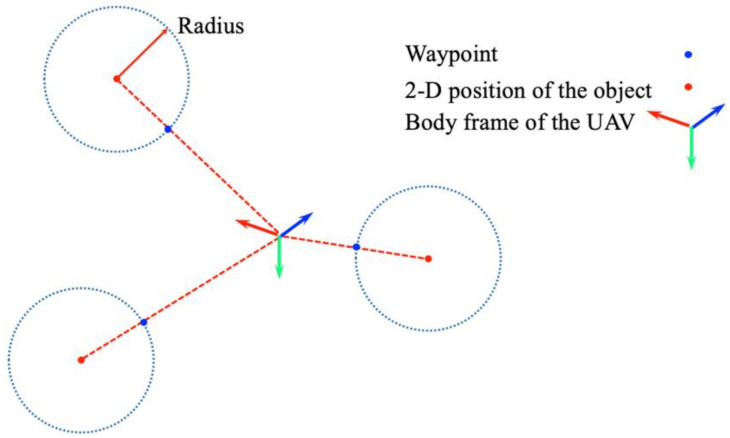
An illustration of waypoints generation. The waypoint (blue) is the intersection point of the circle trajectory and the ray from the vehicle.

To generate a sequence of waypoints for the quadrotor, we consider a two-pronged problem. Generally, aerial photography is necessary for inspection. Therefore, the camera’s optical axis needs to focus on a specific device. As described in the previous section, the circle trajectory is predefined for the UAV to follow. In addition, there are multiple devices. The sequential order when visiting each target device needs to be determined. As such, a geometry method is adopted to generate waypoints for the UAV, connecting the starting point and the target point. Algorithm 2 describes the process of searching for waypoints. To begin, our algorithm is initialized with an empty set M. The UAV position is considered as a 2-D point p. When the 3-D position of the target device $X^{W}$ is obtained by perception, the corresponding 2-D position is represented as $X_{o_{i}}^{W}$. Then, the detected target is represented by a circle with two properties: the 2-D location $\;X_{o_{i}}^{W}$ and the radius r. The **intersect**() function generates a ray from p to $X_{o_{i}}^{W}$ and returns to the ray’s intersection point on $o_{i}$’s circle. After one iteration finishes, the sequence of waypoints is sorted from the shorter one to the farther one by the **sort**() function according to the Euclidean distance between the 3-D object position and the UAV position. Figure 8 provides an illustration of the waypoint generation algorithm.
**Algorithm 2:** Waypoint generation. **Notation**: Object $o_{i}\;$, Object set O, 2-D UAV position p, Waypoint set M, Waypoint 𝓂Initialize: M∈∅**Input:**O, p**for**$o_{i}$ in O𝓂⟵**intersect**(p, $X_{o_{i}}^{W}$)M.**push_back**(𝓂) $o_{i}⟵O$.next()M.**sort**()**return**M

## 6. Experiment Results

The 2-D object detection model deployed on our quadrotor platform is trained by an open-source Darknet framework offline on an Intel Core i5-4690 CPU and two NVIDIA GeForce GRX TITAN Black graphic cards. The trained object detection model is then deployed on a Jetson TX2 onboard computer. All the implementations of the dataset and algorithms in our system have been released for the research community.

The experiments here are designed to demonstrate the following performance aspects:

- The training result of object detection on the prepared dataset including five categories of target devices, demonstrating accuracy and speed performance (Section 6.1).
- Flight experiment with the motion capture system, validating the accuracy of 2-D object detection in the flight test and the 3-D object position estimation (Section 6.2).
- Flight experiment with the VIO localization module, validating the integrated system (Section 6.3).

### 6.1. Training Result of 2-D Object Detection on the Prepared Dataset

The goal of the experiments is to validate the 2-D object detection performance on our prepared dataset (described in Section 4.1.2), which is the prerequisite of 3-D object estimation. The YOLOv4 model was trained over 6000 iterations until the training loss did not decrease any more to prevent overfitting. Then, in the experiment, we compared the model performance with a customized test dataset on different input sizes of 320 × 320, 416 × 416, 512 × 512, and 608 × 608 resolution. Notably, YOLOv4 performed best on our test dataset with a mean average precision with intersection over union threshold of 0.5 ($AP_{50}$) of 98.03% with the input size of 512 × 512 (Table 1). The result has shown that larger input resolutions help increase the mAP (mean average precision (mAP) by increasing the detection precision of the small objects in images. However, for the larger input size of 608 × 608, the mAP shows no significant improvement while the inference speed decreases. Thus, we selected the 2-D detection model with an input size of 512 × 512 as a good trade-off between accuracy and speed.

Some strategies are essential to achieve a good performance of the detection model efficiently. Here are some significant observations from our experiments. First, training the model from scratch helps to reduce false negative results. YOLO allows researchers to save the weights of the model every 1000 iterations and to resume the training from the previously saved model, saving computational time for the training. However, training from scratch helps to reduce the false negative results, compared to training from the previously saved model.

Another observation is that the scale of training data should be kept consistent with the acquired aerial image scale. At the beginning of the experiment, the training dataset consisted of images collected from the internet, where most of the objects cover approximately 70% to 80% of the whole image. However, in our experiment configuration, mAP was not high as expected, since the scale of objects in aerial images captured by UAV only cover 5% to 40% of the whole image.

Lastly, the ratio of target objects to the background image should be close to 1:1 in every training image. For the YOLO detector, objects in the background with similar features will be erroneously predicted as positive examples. In other words, preparing background images in the training set will reduce the false positive (FP) examples increase the model precision.

### 6.2. Flight Experiments in Motion Capture System Environment

**Figure 9:**
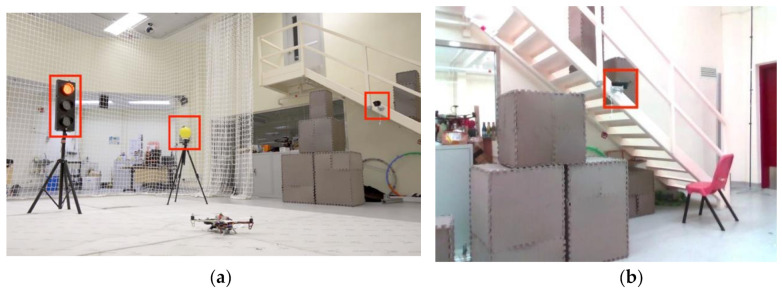
Images of the experiments conducted in the indoor environment. (**a**) Three objects, including a traffic light, a bulb, and a CCTV for inspection mission are marked with red rectangles. (**b**) The target device CCTV is shown outside of the range of the motion capture system.

**Figure 10:**
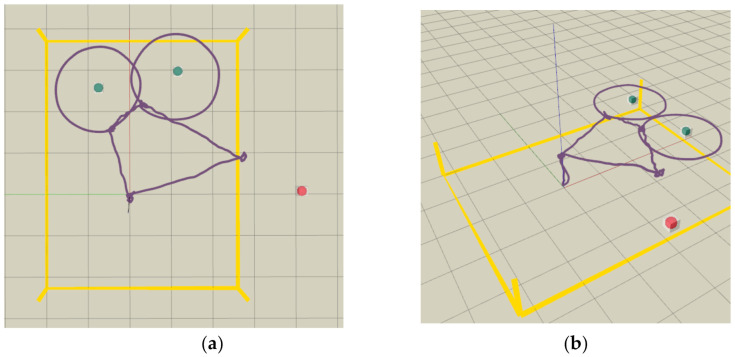
Flight trajectory visualized in RVIZ, shown as the purple curve. The collision status is shown as the red or green sphere, and the boundary of the test site (4.6 × 6.0 m) is shown as yellow lines. (**a**) Bird view (**b**) Side view.

We first conducted flight experiments in a relatively cluttered test site with a motion capture system VICON, to mimic a practical autonomous inspection scenario and validate the robustness of our proposed UAV inspection system. Considering that the locations of three classes of objects (speaker, exit sign, and CCTV) are outside of the VICON’s coverage, three representative devices (a traffic light, a bulb, and a CCTV) are selected to be inspected. Figure 9 depicts the locations of the three devices. The traffic light and bulb have no obstacle behind, while the CCTV is rigidly fixed on the stairs with foam boxes behind. To present flight experiments for inspection application, slow motions for quadrotor are designed. The maximum velocity and acceleration for the quadrotor are set as 0.7 m/s and $0.5\;m/s^{2}$, respectively. The flight trajectory is visualized in Figure 10.

The first goal of the experiments is to evaluate the 2-D object detection accuracy on the quadrotor platform with a real flight. A test dataset is acquired by our visualization module in our quadrotor system, consisting of 1533 aerial images with the resolution of 640 × 480 pixels. The accuracy comparison result is provided in Table 2. We also compared our detection results with the detection method proposed in a real-time UAV warning system. Tijtgat et al. [37] utilized YOLOv2 and a handcrafted feature-based Aggregated Channels Features (ACF) method to detect pedestrians from UAV viewpoint. We can see that YOLOv4 achieved the highest accuracy with AP_50 = 93.23% and input size = 608. In comparison, our 2-D object detection modules achieved better accuracy. Compared with Table 1, the result indicates that small objects can still be recognized by YOLOv4 at a medium distance. Therefore, it can be concluded that our proposed approach effectively and accurately detects target devices in real-world experiments, and we can move on to 3-D position estimation.

**Figure 11:**
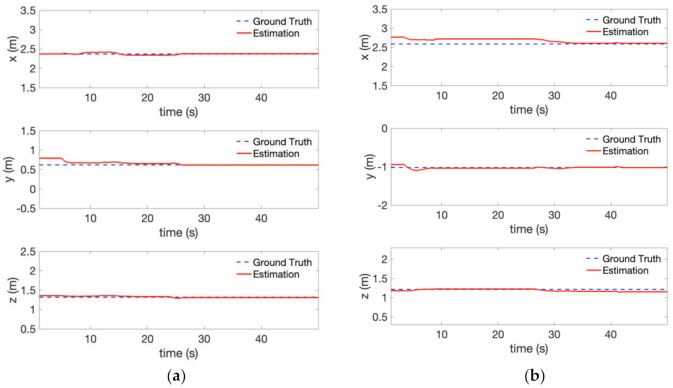
Object position estimation error compared with the ground truth in Figure 10 (localization provided by VICON): (**a**) Traffic light (**b**) Bulb.

To evaluate the 3-D position estimation of the perception module, the estimated object position is compared with the motion capture system (VICON) as the ground truth. Figure 11 compares the 3-D position between the estimation and ground truth. The red dash curve represents the estimated object position filtered by IIR filter, while the blue line shows the ground truth. Generally, we can observe that the estimate of the 3-D object position follows the ground truth. We can also notice the position error caused by the depth measurement, e.g., at the very beginning of the experiment, which could be reduced by decreasing the distance between the camera and the object.

A more specific quantitative evaluation of the 3-D object position estimation is presented using the root mean square error (RMSE) in Table 3. The RMSE is below 0.1 m in all directions. The very low error is partly due to using the ground truth vehicle pose as the input in the object position estimation [7]. Therefore, we reach the conclusion that our proposed method provides an accurate estimate of the target device position and performs an acceptable result, that we can move to visual-inertial odometry experiments.

### 6.3. Visual-Inertial Odometry Flight Experiment

**Figure 12:**
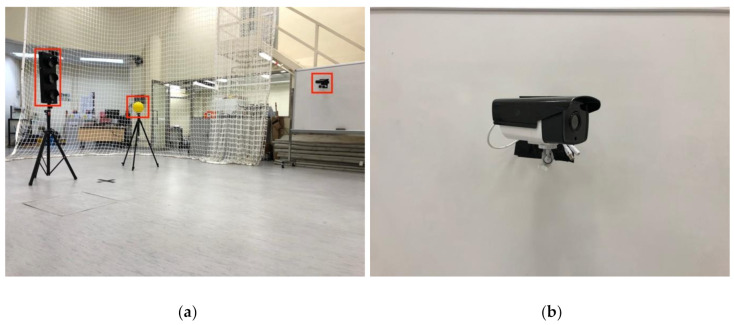
Picture of the visual-inertial odometry flight experiment. (**a**) Three objects: traffic light, bulb, and CCTV for inspection mission are marked with red rectangles. (**b**) The target device CCTV is fixed on a whiteboard with tapes.

**Figure 13:**
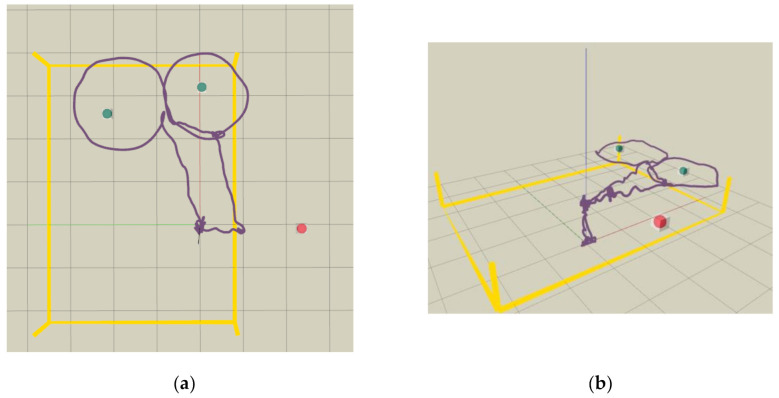
Flight trajectory visualized in RVIZ with the same definitions for the colors used as in Figure 10. (**a**) Bird view, (**b**) Side view.

To further evaluate the performance of the proposed inspection system, we conducted quadrotor flight experiments at the same site with the VIO localization module FLVIS, as shown in Figure 12. No external localization device, such as a motion capture system or GPS, is used to provide the UAV pose. The VICON capture system is only used to monitor the objects’ ground truth state for the later evaluation of our FLVIS method. In this experiment, we changed the background texture of the CCTV with a whiteboard. We also chose another home position to generate a different trajectory, which validates the robustness of our path planning module. Figure 12 depicts the experimental setup in this trial. The flight trajectory is visualized in Figure 13. Details about the experiments can be found in the Supplementary Video.

**Figure 14:**
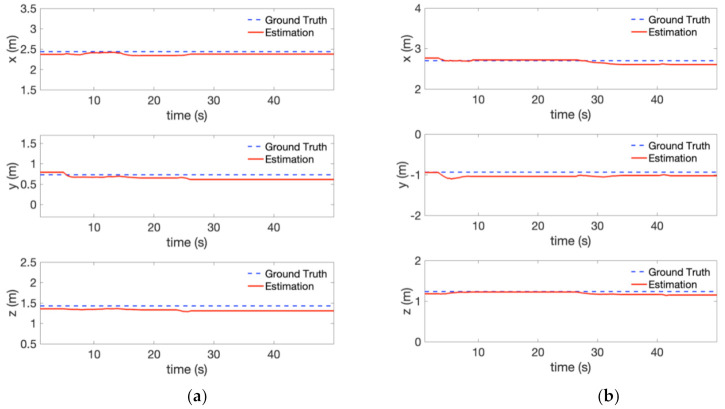
Object position estimation error compared with the ground truth in the visual-inertial odometry flight experiment (localization provided by FLVIS): (**a**) Traffic light, and (**b**) Bulb.

The result of the 3-D object position estimation of this experiment is plotted in Figure 14. The red dash curve and the blue line represents the estimated object position and the ground truth, respectively. The 3-D position estimation follows the ground truth well. However, the error between the estimation and ground truth increased compared with the experiment in Figure 10 (Figure 11). This is partly due to the position drift of vehicle itself caused by visual inertial-odometry. The RMSE for this visual-inertial odometry flight experiment is provided in Table 4**.** The RMSE is below 0.15 m in all directions. In addition, we compared our system with a similar state-of-the-art system [7]. The comparison results are shown in Table 5 using mean absolute error (MAE). For traffic light detection, the mean absolute error using our system is smaller than [7]. For bulb detection, the error in x and z direction also surpasses the baseline in [7] due to the large depth estimation error of stereo camera. It is noticeable that the authors in [7] computed the errors after the hexacopter approached the target tree cavity. By contrast, our system’s large error terms are caused by accumulation through the whole mission procedure, including the approach stage. In consideration of this, our system shows better accuracy in terms of 3-D object position estimation. Hence, it is concluded that our approach can provide relatively accurate estimates of the object position in real-time.

## 7. Conclusions

In this work, we constructed an autonomous UAV inspection system using a learning-based method. We relied on multi-sensor fusion for the localization, object detection, and position estimation of the target device, and path planning for fully autonomous navigation. No prior information about the environment and target devices is required. The feasibility of the proposed system has been proven with accuracy analysis. The experimental results validate our system design and show a satisfactory estimation accuracy of the proposed method.

The current experiments assumed only static objects to be inspected with static obstacles behind. In the future, the path planning module will be further improved to consider moving obstacles in real applications. More outdoor tests will be conducted to test the feasibility and performance of the proposed system widely. We will also continue to develop a globally optimal path planning method considering dynamic obstacles with the help of simultaneous localization and mapping approaches.

## Figures and Tables

**Table 1:** Comparison of accuracy and speed of YOLOv4 on the prepared dataset.

Method	Backbone	Size	$AP_{50}$	FPS
YOLOv4	CSPDarknet-53	320 × 320	94.98%	25
		416 × 416	97.10%	17
		512 × 512	98.03%	13
		608 × 608	98.01%	9

**Table 2:** Comparison of accuracy and speed of yolov4 on the flight test dataset.

**Method**	**Backbone**	**Size**	$AP_{50}$	**FPS**
YOLOv4	CSPDarknet-53	320 × 320	78.04%	27
		416 × 416	90.18%	19
		512 × 512	92.95%	14
		608 × 608	93.23%	10
YOLOv2 [37]	Darknet-19	416 × 416	73.74%	11
		608 × 608	85.94%	5
ACF [37]		40 × 40	79.77%	0.54

**Table 3:** Root mean square error (RMSE) for the estimated object position compared to the ground truth.

Object Category	X (m)	Y (m)	Z (m)
Traffic Light	0.006	0.024	0.003
Bulb	0.071	0.005	0.031

**Table 4:** RMSE for the estimated object position compared to the ground truth.

Object Category	X (m)	Y (m)	Z (m)
Traffic Light	0.062	0.102	0.106
Bulb	0.142	0.093	0.053

**Table 5:** MAE for the estimated object position compared to the ground truth.

Object Category	X (m)	Y (m)	Z (m)
Traffic Light	0.015	0.030	0.016
Bulb	0.071	0.014	0.038
Tree cavity [7]	0.019	0.039	0.024

## Data Availability

The data presented in this study are available in https://github.com/JazzyFeng/LAIS (accessed on 13 February 2021).

## Supplementary Materials

The following are available online at https://youtu.be/OKSm8_4rhzU (accessed on 13 February 2021), Video: Indoor autonomous inspection demonstration. https://github.com/JazzyFeng/LAIS (accessed on 13 February 2021), Source code.
